# Supplementary material for: Emerging Genetic Tools to Investigate Molecular Pathways Related to Heat Stress in Chickens: A Review
Source: Animals (Basel). 2020 Dec 29;11(1):46. doi: 10.3390/ani11010046 (PMC7823582; doi:10.3390/ani11010046)
Supplement: Supplementary file 1 [file animals-11-00046-s001.pdf]

QTL_ID	Chromosome	Coord_A_bp	Coord_B_bp
95395	1	70091023	70992158
95396	1	77003789	77992852
9112	2	0	100
30853	2	121250696	123202074
9114	3	0	100
9426	4	0	100
30862	5	51217211	51587365
9437	6	0	100
30875	11	10785873	11096350
95397	14	3006152	4999182
95401	14	3006152	3999663
95398	15	8001621	8993187
95399	15	8001621	9999305
9123	23	0	100
95400	26	153043	998907
95402	27	1058814	1999774

Table S1. All QTLs related to body temperature in chicken. Coord_A_bp = QTL starting point on a chromosome. Coord_B_bp = QTL ending point on a chromosome. All QTLs are significant.
